# Antimicrobial biopolymer formation from sodium alginate and algae extract using aminoglycosides

**DOI:** 10.1371/journal.pone.0214411

**Published:** 2019-03-26

**Authors:** Lokender Kumar, John Brice, Linda Toberer, Judith Klein-Seetharaman, Daniel Knauss, Susanta K. Sarkar

**Affiliations:** 1 Department of Physics, Colorado School of Mines, Golden, Colorado, United States of America; 2 Department of Chemistry, Colorado School of Mines, Golden, Colorado, United States of America; University of Lisbon, PORTUGAL

## Abstract

Antimicrobial biopolymers provide a biodegradable, sustainable, safe, and cheap approach to drug delivery and wound dressing to control bacterial infection and improve wound healing respectively. Here, we report a one-step method of making antimicrobial alginate polymer from sodium alginate and aqueous extract of Wakame using antibiotic aminoglycosides. Thin layer chromatography of commercially available sodium alginate and Wakame extract showed similar oligosaccharide profiles. Screening of six aminoglycosides showed that kanamycin disulfate and neomycin sulfate produces the highest amount of biopolymer; however, kanamycin disulfate produces the most malleable and form fitting biopolymer. Image texture analysis of biopolymers showed similar quantification parameters for all the six aminoglycosides. Weight of alginate polymer as a function of aminoglycoside concentration follows a growth model of prion protein, consistent with the aggregating nature of both processes. Slow release of antibiotics and the resulting zone of inhibition against *E*. *coli* DH5α were observed by agar well diffusion assay. Inexpensive method of production and slow release of antibiotics will enable diverse applications of antimicrobial alginate biopolymer reported in this paper.

## Introduction

According to the World Health Organization (WHO), the emergence of multidrug resistance among bacterial pathogens is a global public-health challenge [1]. Recently, WHO has listed *Acinetobacter baumannii* [2, 3], carbapenem-resistant *Pseudomonas aeruginosa* [4, 5], and carbapenem-resistant *Enterobacteriaceae* [6, 7] as critical priority pathogens. These pathogens are responsible for infections of burn, wound, blood stream, nervous system, urinary and respiratory tracts. In this context, antimicrobial biopolymer synthesis is one of the current areas of antimicrobial drug delivery research to control bacterial infection in biomedical devices [8], wound healing [9], food packaging [10], textiles [11], cosmetic products [11], and water treatment systems [12]. Furthermore, antimicrobial biopolymers are safe, less toxic and more efficacious as compared to the low molecular weight quaternary ammonium compounds [13]. While some polymers have intrinsic antimicrobial activity [14], others have antimicrobial compounds attached to the polymer backbone [15] or trapped within the polymer [16]. Antibiotics can be trapped during the polymerization reaction or by loosely bound cleavable linkages [17].

Long chain polysaccharides such as alginate are particularly suitable for polymer synthesis due to their biocompatibility, biodegradability, low cost, and non-toxicity to human cells [18]. These properties cover a wide variety of uses in the biomedical and food industries. While chitosan and polylysine are widely known biopolymers with intrinsic antimicrobial properties [19, 20], alginate and other polysaccharides such as cellulose are not intrinsically antimicrobial in nature and require chemical incorporation of an antibiotic moiety. Alginate, in particular, has gained interest because it is a sustainable polymer present in the cell walls of brown algae (*Phaeophyceae*), including *Undaria pinnatifida*, *Laminaria digitata*, *Laminaria hyperborea*, *Laminaria japonica*, *Macrocystis pyrifera*, and *Ascophyllum nodosum* [21]. Alginate constitutes more than 50% of dry weight as lignin-free carbohydrate in Wakame (*Undaria pinnatifida*), a popular edible brown algae native to the Pacific Ocean and one of the 100 most invasive species in the world [22]. In general, alginate has found applications in drug delivery [23], wound healing [24], and tissue engineering applications [25] due to its biocompatibility, biodegradability, and ease of gelation [26].

Aminoglycosides have been an important class of antibacterial drugs, especially for the treatment of Gram negative bacterial infections [27]. These drugs show efficient post antimicrobial effects against pathogenic bacteria and maintain prolonged activity [28]. Aminoglycosides target the protein translation machinery and bind reversibly to the bacterial 30s ribosomal subunit, causing misreading of the genetic code and accumulation of non-functional truncated proteins leading to the death of bacteria [29]. Aminoglycosides also show potential ototoxicity and nephrotoxicity; therefore, slow drug release using advanced drug delivery methods is clinically important [30]. Embedding aminoglycosides in biopolymers such as hydroxypropyl methylcellulose (HPMC)/xyloglucan (XG) loaded with gentamicin sulfate has shown high potency and thermal stability [31]. Similarly, the integration of gentamicin sulfate with crosslinked collagen and sodium alginate crosslinked with Ca^2+^ ions allowed controlled delivery of the antibiotic in treatment of post-operative bone infections [32]. Tobramycin-alginate/chitosan polymeric nanoparticles (NPs) were shown effective in treatment of *P*. *aeruginosa* infections [33]. Neomycin sulfate-loaded polyvinyl alcohol (PVA), polyvinyl pyrrolidone (PVP), and sodium alginate (SA) dressing enhanced wound healing [34]. In general, antimicrobial biopolymers made from polysaccharides are considered superior to polymers made from synthetic polyesters or polyacrylic acid since these synthetic polymers have a risk of toxicity and a high cost of synthesis [35]. In this context, the carbodiimide chemistry is a viable strategy to chemically attach aminoglycosides to polysaccharides and has been used to gentamycin to alginate [15] and chitosan [36]. However, the carbodiimide chemistry involves several steps, making it relatively expensive. Additionally, there is irreversible attachment of the aminoglycosides preventing release.

In this paper, we demonstrate a one-step method of making antimicrobial alginate polymer using aminoglycosides that is inexpensive and enables a slow release of antibiotics. We screened gentamicin sulfate (GS), neomycin sulfate (NS), kanamycin sulfate (KS), kanamycin disulfate (KDS), tobramycin sulfate (TS), and streptomycin sulfate (SS) for polymerization efficiency and antimicrobial activity of the synthesized polymer. Both an aqueous extract of *Undaria Pinnatifida* (Wakame) and aqueous solution of sodium alginate (SA) showed similar polymerization behavior. We modeled the growth of the alginate polymer analogous to that of pathogenic prion protein. Slow release of antibiotic, possibly due to thermal breathing, led to clear zones of inhibition against *E*. *coli* DH5α in agar diffusion assay. This study provides an enabling methodology for further research utilizing alginate/aminoglycoside polymers on antimicrobial wound dressings, artificial skin tissue, artificial antimicrobial blood clot agent, food packaging system, cosmetics, and waste water treatment systems.

## Results

### Oligosaccharides detection using TLC, quantification of polymerization efficiency, and quality of alginate polymers

**Fig 1:**
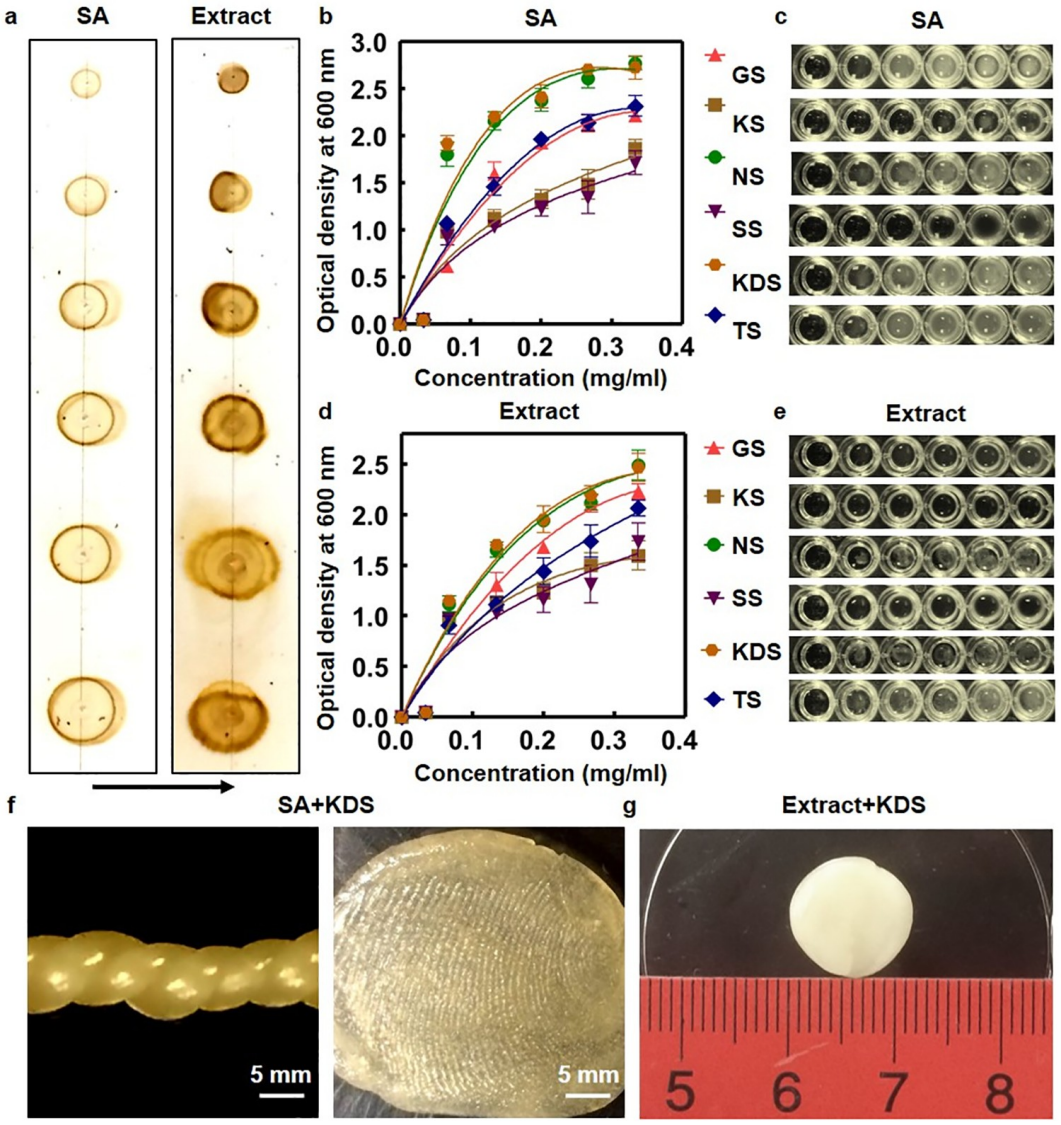
Quantification of efficiency of aminoglycosides in alginate polymerization. (**a**) TLC of commercial SA and Wakame extract show oligosaccharide profiles; the black arrow shows the flow direction of the mobile phase. (**b**) Optical density of commercial SA solution for different aminoglycosides; GS, KS, NS, SS, KDS, and TS. (**c**) Image of wells in a microtiter plate for commercial SA solution and different aminoglycosides; white turbidity led to higher optical density values. (**d**) Optical density of Wakame extract after addition of different aminoglycosides. (**e**) Image of wells in a microtiter plate for Wakame extract solution and different aminoglycosides. (**f**) Malleability and form fitting alginate biopolymer using KDS. Malleability and form fitting alginate biopolymer using KDS after kneading; twisted strands (left) and fingerprint capture (right). (**g**) Biopolymer made from Wakame extract using KDS.

We used TLC to check the oligosaccharide profiles [37] of commercial SA and aqueous Wakame extract (Fig 1a). Similar TLC profiles confirm the similarities of oligosaccharide compositions of SA and Wakame extract. For quantifying polymerization efficiency, we measured OD at 600 nm using a plate reader to quantify turbidity due to alginate polymerization to screen six aminoglycosides (Fig 1b and 1d). Optical density (OD), affected by both scattering and absorption, is a common technique to quantify cell growth, fluorophore concentration, and turbidity due to suspended particles [38]. OD of the polymerization reactions at varying concentrations of aminoglycosides showed initial growth followed by saturation. We fitted the OD measurements to *y* = *ax* / (*k* + *x*), a general equation describing ligand binding to substrate where *k* is the binding affinity [39]. Fit parameters for aqueous solution of sodium alginate (Fig 1b) are: *a* = *3*.*66* ± *1*.*66*, *k* = *0*.*19* ± *0*.*18* (GS); *a* = *2*.*77* ± *1*.*55*, *k* = *0*.*20* ± *0*.*25* (KS); *a* = *3*.*10* ± *0*.*38*, *k* = *0*.*05* ± *0*.*03* (NS); *a* = *2*.*11* ± *1*.*01*, *k* = *0*.*12* ± *0*.*17* (SS); *a* = *3*.*52* ± *0*.*62*, *k* = *0*.*17* ± *0*.*07* (TS); *a* = *3*.*06* ± *0*.*37*, *k* = *0*.*04* ± *0*.*03* (KDS). Fit parameters for aqueous Wakame extract (Fig 1d) are: *a* = *4*.*00* ± *1*.*35*, *k* = *0*.*26* ± *0*.*16* (GS); *a* = *2*.*04* ± *0*.*67*, *k* = *0*.*10* ± *0*.*08* (KS); *a* = *3*.*59* ± *0*.*78*, *k* = *0*.*16* ± *0*.*09* (NS); *a* = *2*.*62* ± *2*.*34*, *k* = *0*.*22* ± *0*.*42* (SS); *a* = *3*.*28* ± *1*.*61*,*k* = *0*.*23* ± *0*.*89* (TS); *a* = *3*.*48* ± *0*.*60*, *k* = *0*.*15* ± *0*.*07* (KDS). We used *k* as the measure of efficiency of aminoglycosides in polymerizing alginate. Neomycin and kanamycin disulfate resulted in the best polymerization. We selected KDS for further testing because it is relatively inexpensive and made alginate biopolymer from SA and Wakame extract, both of which resulted in good quality polymer (Fig 1f and 1g); however, SA solution provided more malleable (Fig 1f, left panel) and form fitting (Fig 1g, right panel) polymer.

### Melting temperature of polymer

**Fig 2:**
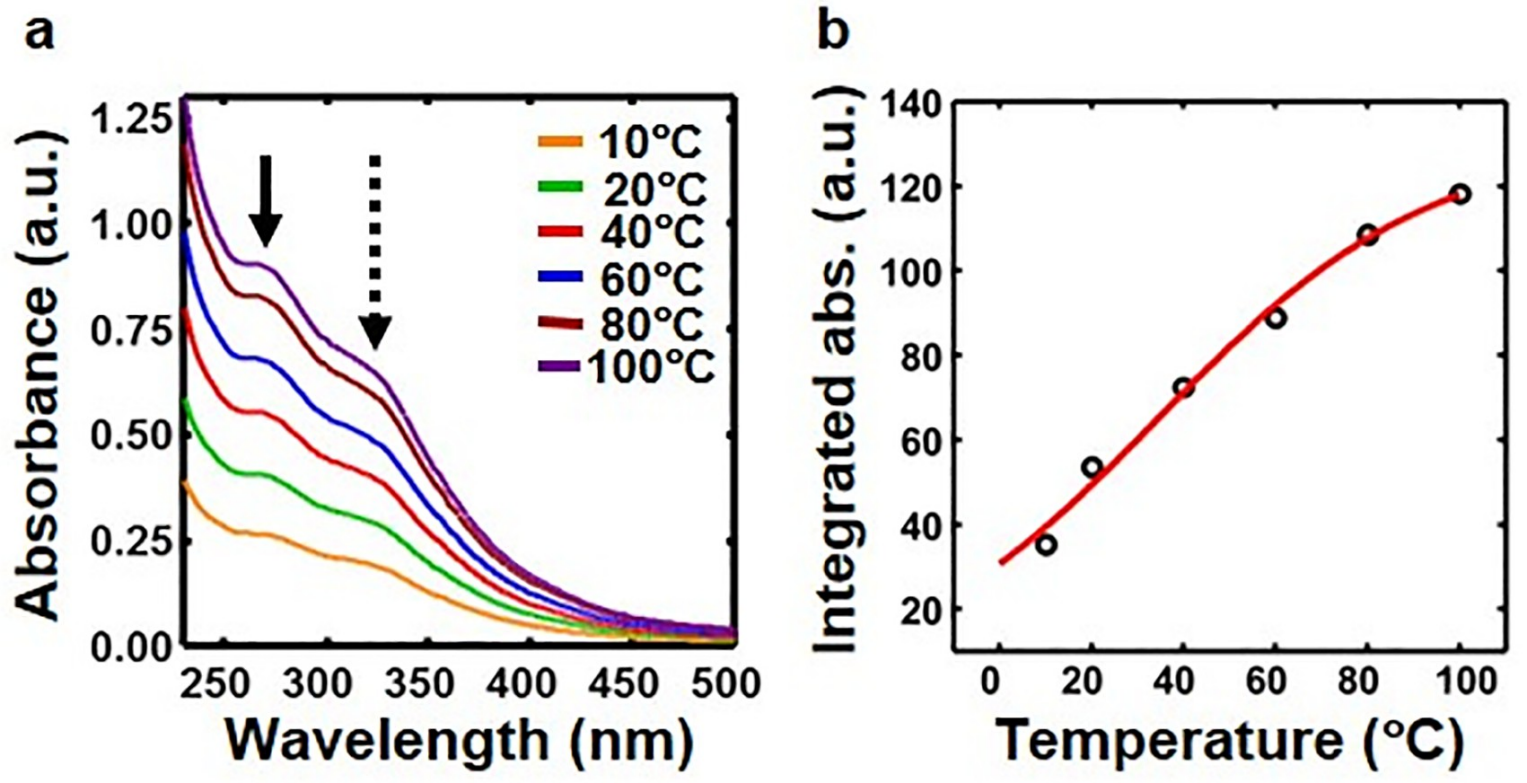
Melting of antimicrobial alginate polymer. (**a**) Absorption spectra of the centrifugation supernatant after incubation of polymer in 1 M NaOH solution for 30 min at different temperatures. The peaks due to alginate and kanamycin disulfate are indicated by the solid and dotted black arrows respectively. (**b**) Integrated absorption (open black circle) as a function of temperature fits well to a sigmoidal function (red line) with a melting temperature of 34.7±2.5°C.

We measured the melting temperature by leveraging temperature-dependent dissolution of alginate polymer. Polymer was incubated in 1 M NaOH solution to break ionic bonds in polymer and dissolve. Reaction was centrifuged and absorption spectrum of the supernatant was measured. Experiment was repeated at different temperatures (Fig 2a). Integrated absorption, i.e., area under the absorption spectrum, as a function of temperature (Fig 2b) was fitted to a sigmoidal function, *y* = *a* / (1 + exp(*T*_*m*_ − *T*) / *σ*), where *T*_*m*_ is the melting temperature and *σ* is the width. Fit parameters are: *a* = 130.8 ± 4.3(*std*), *T*_*m*_ = 34.7 ± 2.5(*std*), and *σ* = 29.4 ± 2.1(*std*).

### Microscopy to visualize and quantify polymer texture

**Fig 3:**
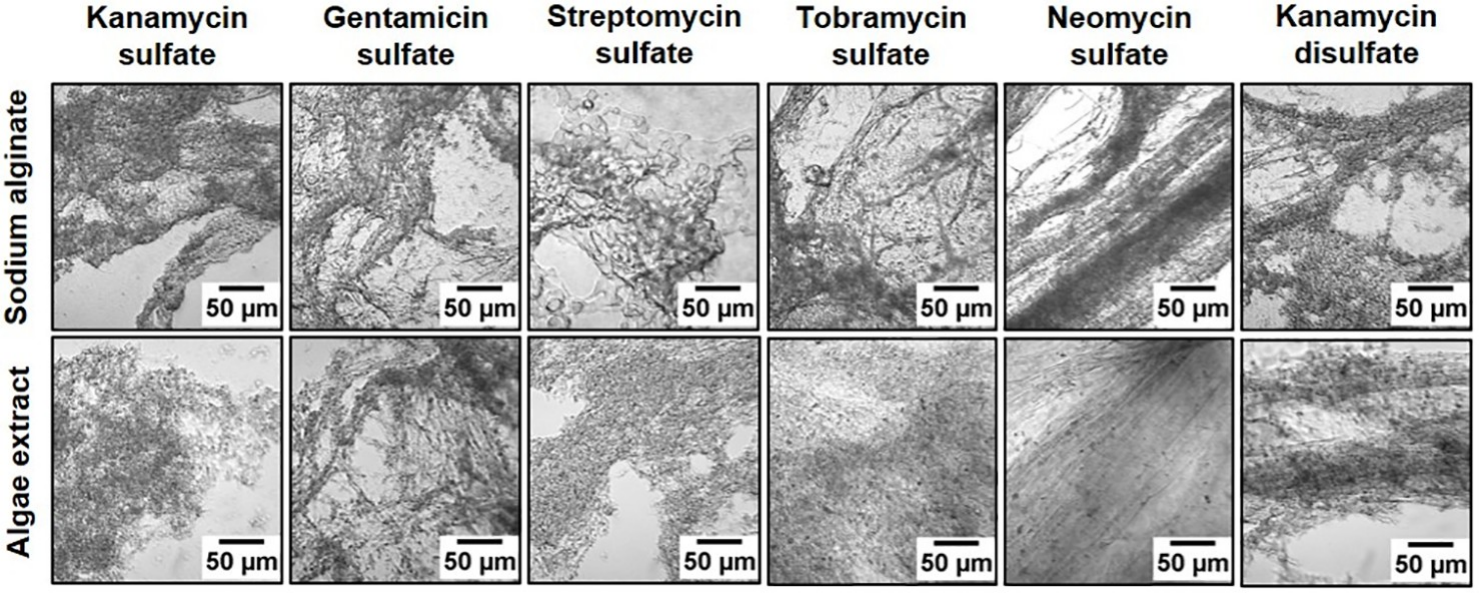
Texture of alginate polymers. Addition of aminoglycoside to sodium alginate and algae extract resulted in more thread-like polymers for neomycin sulfate and kanamycin disulfate, whereas other aminoglycosides showed more granular polymers.

**Fig 4:**
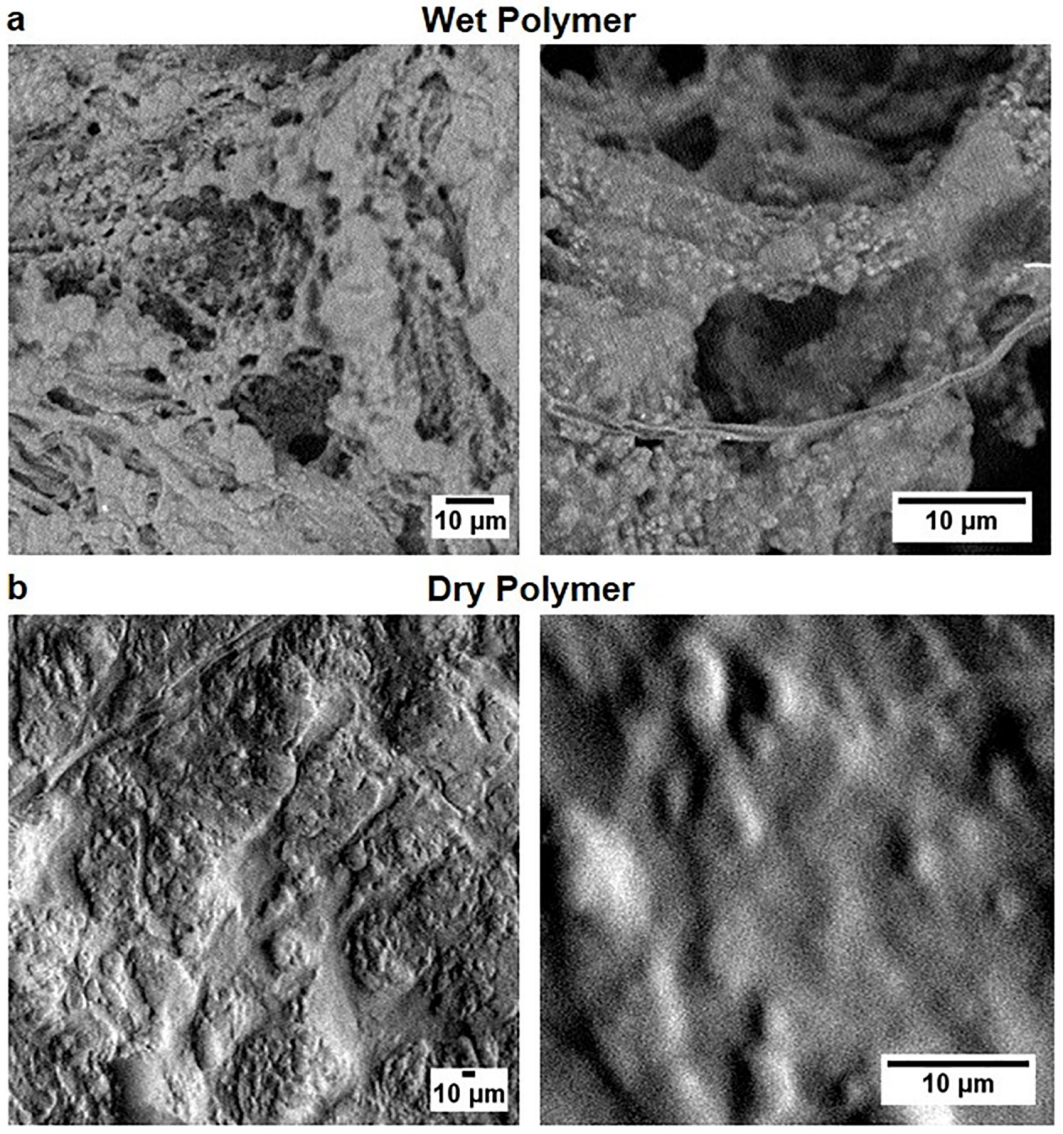
Scanning electron microscope (SEM) imaging of polymer. (**a**) Images of wet polymer immediately after formation at low resolution (left panel) and high resolution (right panel) show threads and porous structures. (**b**) Images of dry polymer at low resolution (left panel) and high resolution (right panel) show a lack of porous structures observed in wet polymer.

To visualize the texture of alginate polymers, we performed reactions on microscopes slides and imaged using an optical microscope at 10X magnification as shown in Fig 3. Since biopolymer texture can affect surface reactions and absorption of ligands [40], quantitative analysis of polymer is useful and has been reported for alginate and chitosan films [41]. To avoid subjective bias in visual observation, we quantified the polymer texture using four quantitative parameters (energy, E; contrast, C; homogeneity, H; and entropy, S) of Gray Level Co-Occurrence Matrix (GLCM) algorithm [42] and one quantitative parameter (fractal dimension, F) of Shifting Differential Box Counting (SDBC) algorithm [43, 44] (see Methods for definitions and detailed procedures). These five parameters have been previously used for texture analysis of alginate gel images [41]. We calculated E, C, H, S, and F for nine 640 pixel × 480 pixel images for each condition. Table 1 shows the quantitative parameters for different aminoglycosides. To determine polymer texture at higher resolutions, we imaged KDS-based alginate polymer using SEM (Fig 4). Wet polymer is porous (Fig 4a), whereas dry polymer is not porous (Fig 4b).

**Table 1:** Quantitative parameters of alginate polymer texture.

Antibiotic	Commercial alginate					Algae extract				
	E	C	H	S	F	E	C	H	S	F
GS	0.0003±0.0002	439.1±172.4	0.16±0.05	9.1± 0.2	0.23±0.07	0.0003±0.0002	282.1±84.4	0.17±0.04	8.8± 0.4	0.27±0.06
KS	0.0003±0.0001	344.4±68.3	0.17±0.02	8.9± 0.2	0.27±0.04	0.0007±0.0003	253.1±94.5	0.23± 0.05	8.3± 0.5	0.30±0.05
NS	0.0034± 0.0048	608.8±188.7	0.17±0.05	9.2± 0.5	0.20±0.05	0.0005±0.0001	114.7±48.0	0.25±0.03	8.1± 0.2	0.36±0.04
SS	0.0003±0.0001	430.6±116.5	0.17± 0.03	9.1± 0.3	0.21±0.03	0.0004±0.0001	384.2±43.18	0.18±0.02	8.9± 0.1	0.23±0.03
TS	0.0009±0.0001	316.1±61.0	0.20± 0.03	9.0± 0.3	0.28±0.03	0.0003±0.0001	201.3±45.2	0.18±0.04	8.6± 0.3	0.31±0.04
KDS	0.0005±0.0004	470.3±13.9	0.17±0.03	9.2± 0.2	0.21±0.02	0.0003±0.0001	230.3±106.1	0.18±0.04	8.8±0.3	0.29±0.05

### Dry weight of pellet after centrifugation of polymerization reaction to quantify the total amount of polymer

**Fig 5:**
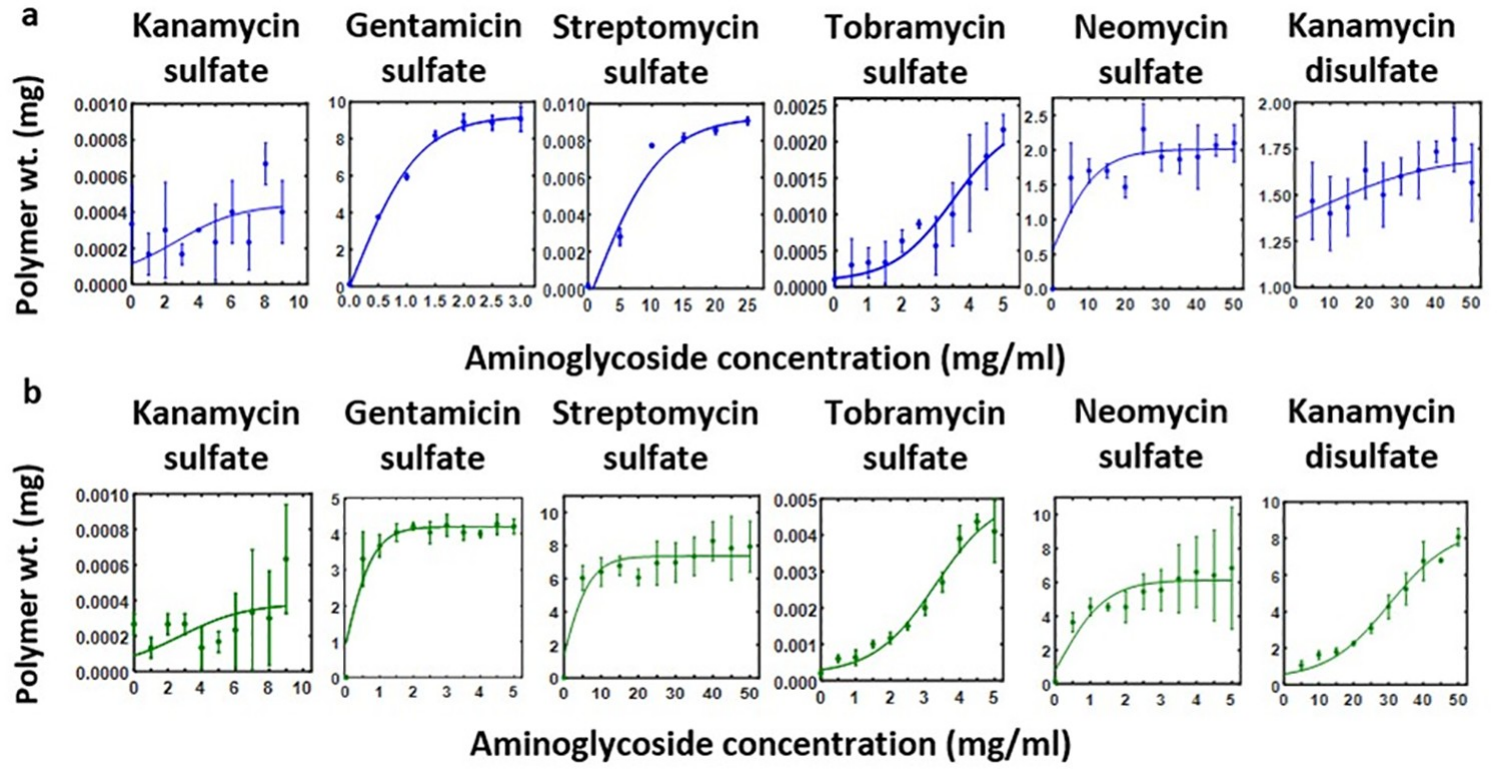
Quantification of polymer formation for different aminoglycosides and prion protein-like growth model. Different concentrations of aminoglycoside were added to (**a**) sodium alginate and (**b**) Wakame extract. The solution was centrifuged and dried to measure the weight of polymer.

Quantitative weight-based assay approach was used to quantify the optimum amount to antibiotic and sodium alginate for polymer synthesis. Different concentrations were tested with a fixed concentration of sodium alginate and algae extract. To quantify the total amount of alginate polymer created after adding aminoglycosides to alginate solutions, the reactions were centrifuged. Supernatants were decanted and pellets were dried. Dried polymer weights were measured for different concentrations of aminoglycoside (Fig 5). We fitted the experimental polymer weights against concentrations (Fig 5) to a model similar to prion protein growth [45]. Best fit parameters (Table 2) were used to calculate the dry weight of alginate polymer per mg of antibiotics.

**Table 2:** Fit parameters for prion protein-like growth model of alginate polymer and dry weight, *w* (mg) of polymer per mg antibiotic.

Antibiotic	Commercial alginate					Algae extract				
	*b*	*n*	*r1*	*r2*	*w*	*b*	*n*	*r1*	*r2*	*w*
GS	0.07	1	0.59	-0.62	6.3578	0.38	1	0.92	-1.85	3.6457
KS	1266	0.5	0.79	-0.42	0.0003	878.9	0.5	0.18	-0.48	0.0003
NS	0.20	1	0.20	-0.36	0.6861	0.04	1	0.26	-0.24	4.0841
SS	50.74	0.5	0.39	-0.48	0.0002	0.05	1	0.23	-0.38	2.2524
TS	161.4	0.5	0.12	-0.43	0.0002	61.23	0.5	0.06	-0.42	0.0005
KDS	0.28	1	0.32	-0.29	1.0993	0.07	3.7	0.21	-0.25	0.7823

### Measurement of zone of bacterial inhibition to quantify antimicrobial property of alginate-aminoglycoside polymer against *E*. *coli* DH5α

**Fig 6:**
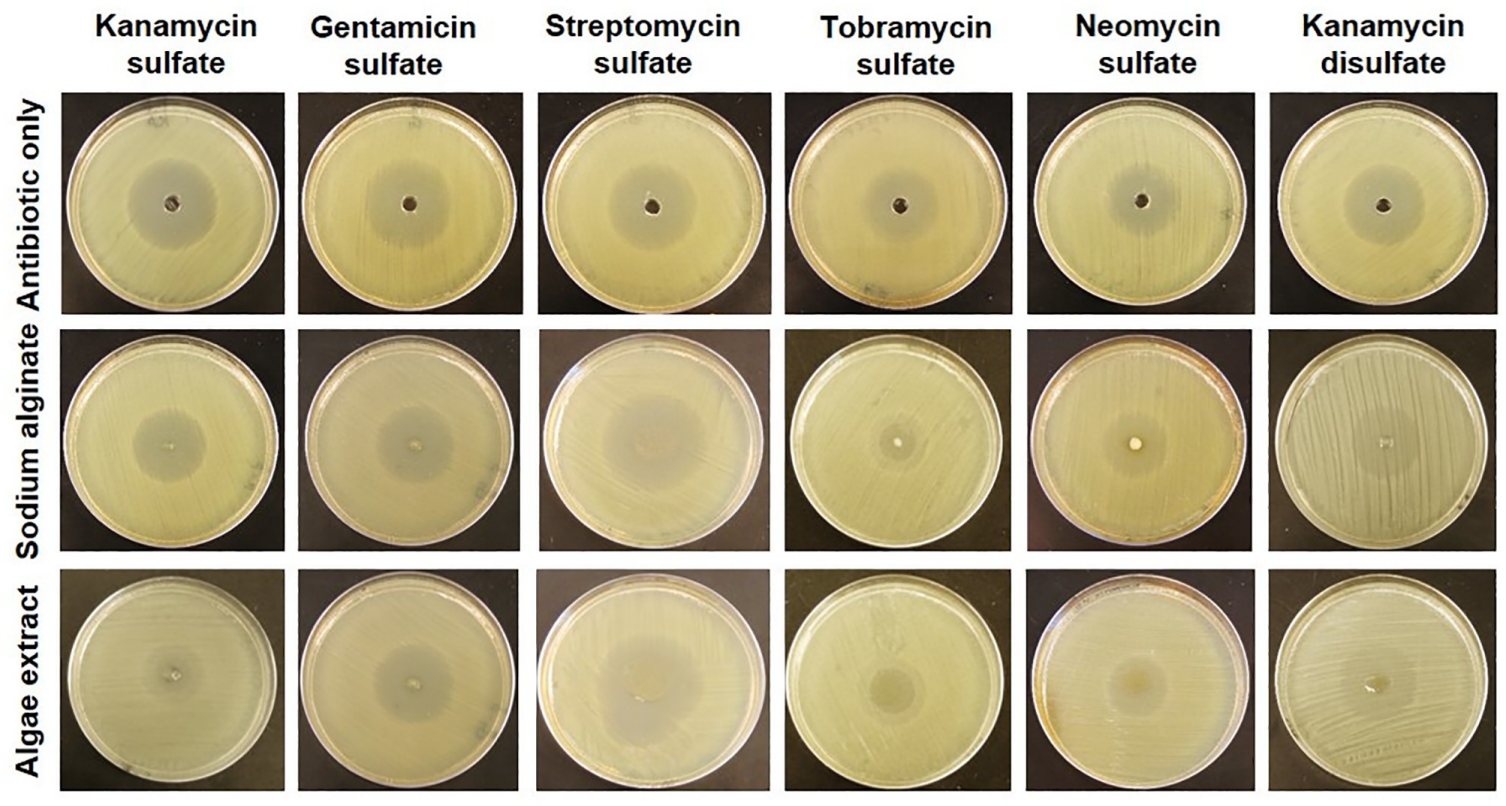
Antimicrobial activity of biopolymers against *E*. *coli* DH5α. Circular zones of growth inhibition after antibiotics and polymers were placed in wells at the centers of LB plates.

Aminoglycoside biopolymers were prepared in microcentrifuge tubes and after centrifugation, pellet was washed three times with sterile deionized water to remove any free antibiotic. After washing, the polymer pellet was carefully removed using a spatula and placed at the center of the plate containing *E*.*coli* DH5α cells and incubated for 18 hr. Circular zones of inhibition were observed against *E*. *coli* DH5α strain **(**Fig 6**)**. After incubation for 18 hr, white polymer pellets became transparent to varying degrees. Higher degree of transparency after incubation generally resulted in large zone of inhibition. While polymers made with KS, GS, and KDS became transparent, polymers made with TS and NS remained white. Alginate polymer made using SS was the most fragile and resulted in the largest zone of inhibition. The diameters of zone of inhibition for different antibiotics are given in Table 3.

**Table 3:** Diameter (cm) of antimicrobial zone of inhibition by biopolymer against *E*.*coli* DH5α.

Antibiotics	Commercial alginate	Algae extract	Antibiotic only
GS	2.6±1.3	2.9±0.1	2.9±0.1
KS	3.7±0.1	3.5±0.2	3.4±0.1
NS	2.9±0.1	3.0±0.2	3.0±0.2
SS	4.2±0.1	3.8±0.1	3.5±0.1
TS	2.6±0.1	3.0± 0.1	3.1±0.1
KDS	3.0±0.2	3.0±0.2	3.0±0.1

### Biocompatibility assay

**Fig 7:**
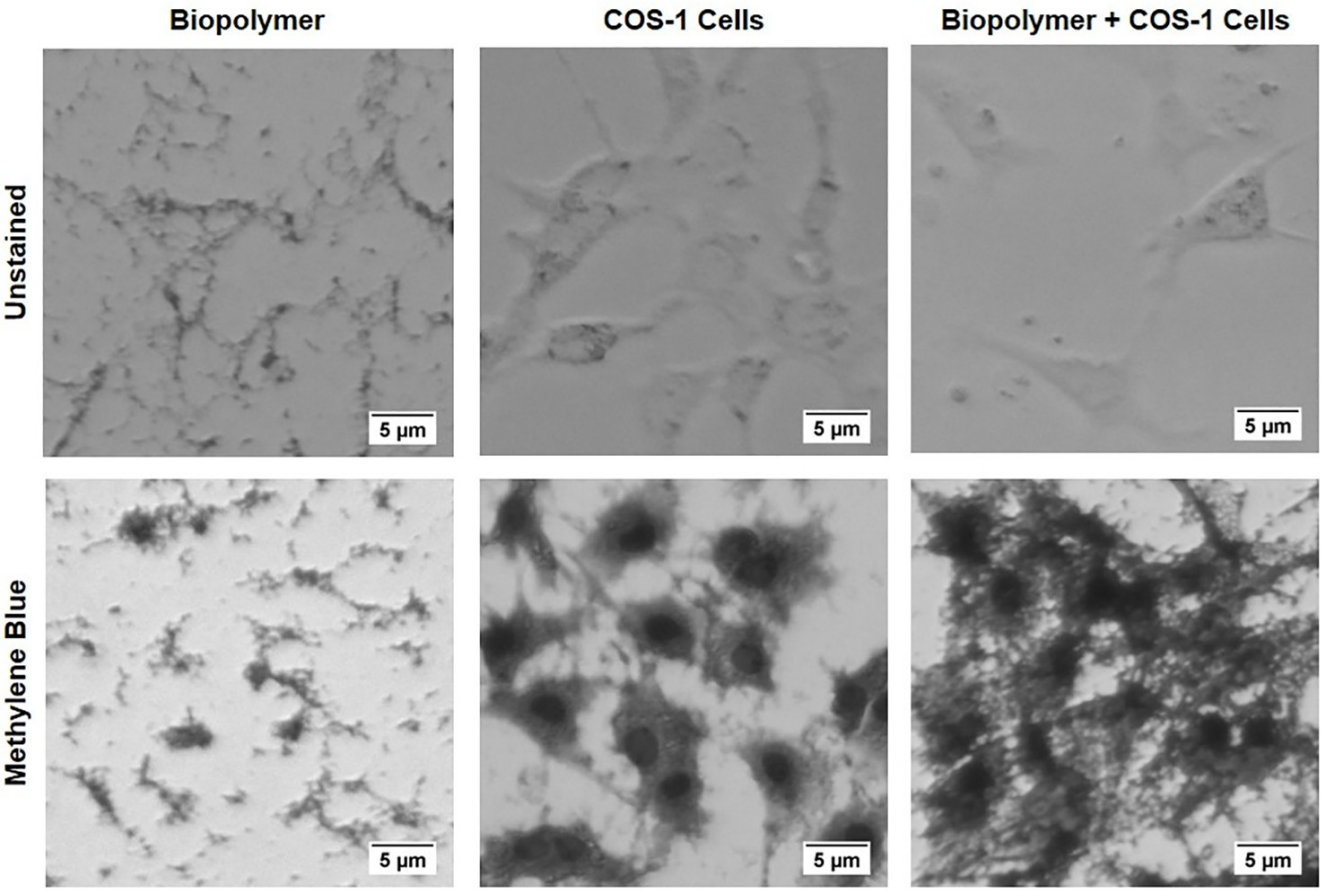
Biocompatibility of alginate polymer. Unstained (top panels) Fluorescent images (8 bit grayscale) of cell culture plates with biopolymer only (top left panel), COS-1 cells only (top middle panel), and COS-1 cells on biopolymer (top right panel). Staining with methylene blue increases the contrast (bottom panels). Methylene blue preferentially stains cell nuclei and provides more contrast. COS-1 cells grows on and attach to alginate polymer indicating biocompatibility.

We grew COS-1 cells on alginate polymer. As shown in Fig 7, COS-1 cells attached, grew, and formed a nest of polymer. Presence of viable cells clearly indicated that aminoglycoside-based alginate polymer is not toxic to COS-1 cells.

## Discussion

### Sodium alginate polymerizes due to acid-base reaction mechanism

Algae extract and commercially available sodium alginate showed similar polymerization behaviors with aminoglycosides. The most likely mechanism of polymerization is interactions between sodium ions in alginate polymer and sulfate ions in aminoglycoside antibiotics. Immediately after addition of aminoglycoside in sodium alginate, the sulfate ions bind the sodium ions to form sodium sulfate and aminoglycoside binds to alginate via amine-carbonyl interactions. The addition of polymer in sodium hydroxide solution reversed the interaction, which was proved by adding sodium hydroxide in polymer pellet followed by heating for 30 min (Fig 2).

### Polymerization efficiency depends on the type of aminoglycoside

We considered the cost of aminoglycosides and the amount of alginate polymer produced using 1 mg of aminoglycoside to determine the efficiency of polymerization, which follows the sequence GS>NS>SS>TS>KS>KDS in decreasing order of efficiency. However, KDS results in the most malleable and form fitting polymer. According to the mechanism of polymerization described before, both the number of amines and sulfates should affect polymerization. While there is a general trend to support the importance of amines and sulfate, the order does not follow exactly, which suggests other factors in polymerization that we do not know yet.

### Growth of alginate polymer is similar to a model of prion protein growth

In general, alginate undergoes complex hierarchical crosslinking and aggregation [46] typical of polysaccharides [47]. To simplify, we modeled alginate polymerization [46] similar to the pathogenic form of prion protein [45] because both are unbranched linear polymers and form stable aggregates. Experimentally, we waited 30 min to account for the initial time-dependent polymerization so that the total amount of time-independent alginate polymer can be described by[45]:

$$y=n-\frac{1}{2}+\frac{r1-r2}{2b}\times \text{tanh}\left[\frac{r1-r2}{2}x+\frac{1}{2}\text{ln}\left(\frac{\left(r1-r2)+b(1-2n+2y(0\right)}{\left(r1-r2)-b(1-2n+2y(0\right)}\right)\right]$$

where *y* is the total alginate polymer, *y(0)* is the initial size of alginate polymers, *n* represents the minimum size of stable alginate polymers, *r1* and *r2* are the growth and dissociation rates of alginate polymers, and *b* is the breakage rate of a alginate polymeric chain. As shown in Fig 5, this model fits polymer weights well as a function of concentrations for all the aminoglycosides.

### Heat-induced dissociation results in slow release of aminoglycoside from alginate biopolymers

**Fig 8:**
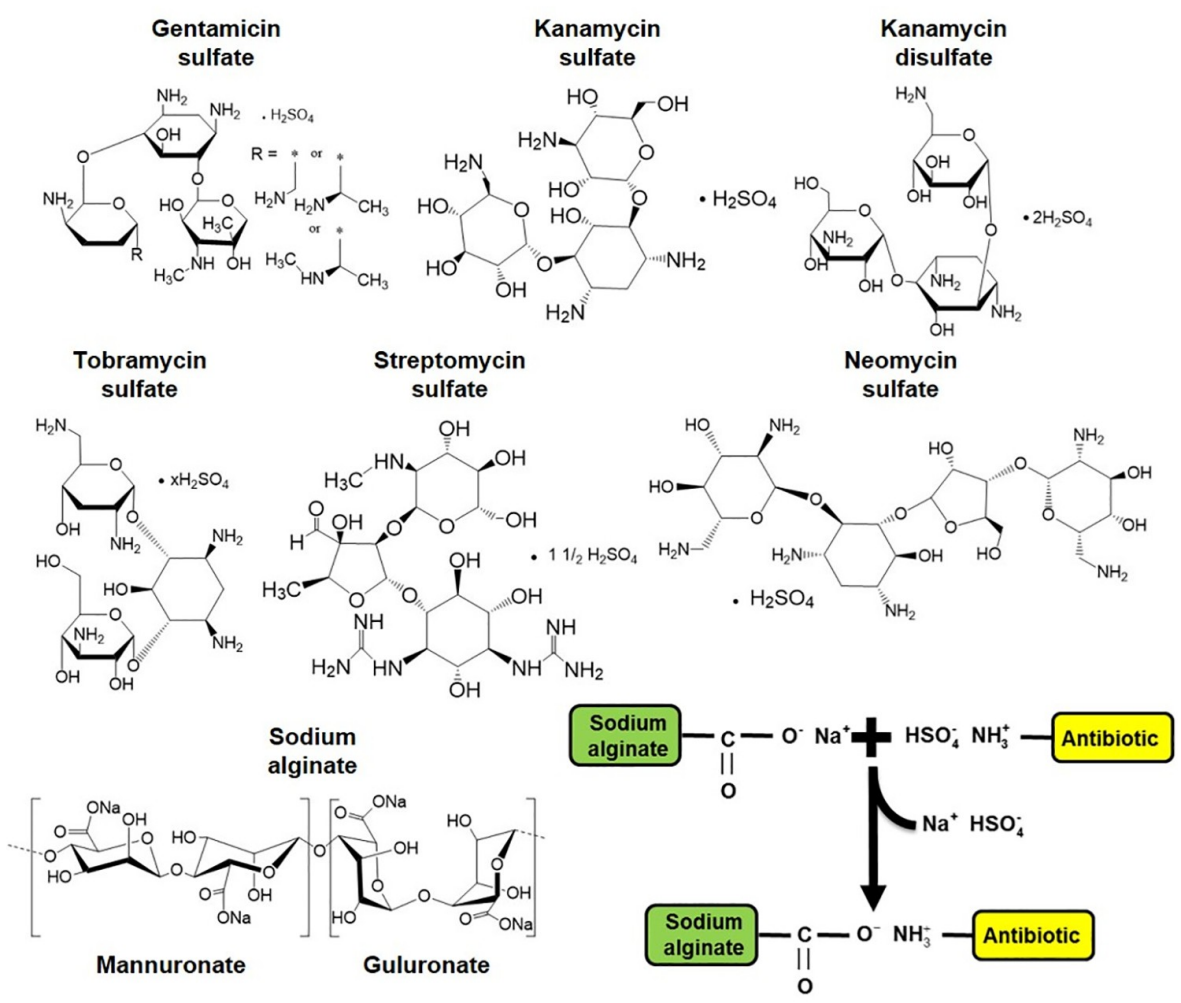
Mechanism of polymerization. Chemical structures of aminoglycosides and mechanism of aminoglycoside-based alginate polymer.

Analogous to hydrogen bonds in double-stranded DNA, we posit that aminoglycoside-based sodium alginate polymer is held together by ionic bonds as a result of acid-base reaction. Therefore, bonds in alginate polymer are expected to break in NaOH solution in a temperature-dependent manner as shown in Fig 2. Indeed, the measured melting temperature *T*_*m*_ = 34.7 ± 2.5(*std*) of alginate polymer (Fig 2b) is of the same order as that of DNA. Since the alginate polymerization occurs due to electrostatic interactions in water (Fig 8), the bond strength is similar to hydrogen bonds [46]. As such, thermal breathing of bonds between aminoglycoside and alginate is possible similar to the thermal breathing of hydrogen bonds in DNA [48]. Thermal breathing along with any base in the media that can create competing ions provides a plausible mechanism of the slow release of aminoglycoside from alginate biopolymers. Additionally, when a piece of aminoglycoside-based alginate polymer is placed at the center, aminoglycosides stochastically may detach from alginate due to thermal breathing and quickly diffuses away due to the concentration gradient. Since streptomycin sulfate has only two amines, it detaches easily from alginate polymer and leads to highest zone of inhibition (Fig 6). In contrast, neomycin sulfate has six amines, which leads to more chance of attachment after detachment due to thermal breathing. As a result, neomycin sulfate provides more stable alginate polymer and slower release leading to smaller zone of inhibition (Fig 6). Release time can possibly be controlled by the number of sulfate ions in aminoglycosides because an argument similar to the number of amines can be made regarding more reversible detachment with more sulfate ions. However, further studies are needed to confirm these hypotheses.

### Aminoglycoside-based alginate polymer as potential niche for tissue engineering

Alginate is an well-established naturally derived alternative to synthetic hydrogels such as polyethylene glycol (PEG) and acts as an excellent extracellular mimics to provide the biological cues to cells and surrounding tissue [49]. Both alginate and alginate functionalized with an RGD peptide (RGD-Alginate) with enhanced cell adhesion have been used as extracellular matrix analogs for tissue engineering [50–60]. Flexible (Fig 1f and 1g), porous (Fig 4a), and biocompatible (Fig 7) aminoglycoside-based alginate polymer developed in this paper provides an easy alternative niche for tissue engineering without added chemicals.

## Conclusion

In conclusion, we have described a method of preparing biocompatible antimicrobial alginate polymer from aqueous solution of commercial sodium alginate and aqueous extract of Wakame using aminoglycoside antibiotics. The underlying acid-base mechanism involves interactions between negatively charged oxygen due to dissociated sodium ions in alginate and protonated amine in aminoglycosides. Polymerization efficiency seems to loosely correlate with the number of amines and sulfate ions in aminoglycosides. Slow release of aminoglycosides from alginate polymers is evident from the microbial zone of inhibition. Antimicrobial alginate polymers from Wakame, one of the most invasive species in the world that grows in diverse conditions of vast oceans, provides a sustainable and biodegradable alternative for wound dressing with slow release of antibiotics.

## Methods

### Alginate extraction from Undaria pinnatifida (Wakame) using mechanical grinding and orbital shaking

Commercially available dry Wakame algae leaves (Amazon.com) were converted into powder using a coffee grinder. 20 g of dry powder was added to 500 ml water in an Erlenmeyer flask and incubated at 37°C for 15 hr with 250 rpm orbital shaking. Following incubation, aqueous sodium alginate supernatant was collected after centrifugation at 10000 rpm for 10 min. All steps were performed under sterile conditions to avoid microbial contamination. The supernatant was used for making alginate polymers using aminoglycoside antibiotics.

### Thin layer chromatography (TLC)

Sodium alginate and algae extract were spotted on TLC plates (Millipore, Cat# Hx71642853, TLC silica gel, 60 aluminum sheets, 20×20 cm) at several places using 5, 10, 15, 20, 25, and 30 μl of solutions. TLC plates were developed using a mixture of 1-butanol, formic acid, and water in 4:6:1 (v:v:v) ratio. The developed TLC plates were heated at 110°C for 5 min after spraying with 10% (v/v) sulfuric acid in ethanol to test for the presence of alginate oligosaccharides [37].

### Screening of aminoglycosides for polymerization reaction using optical density measurements

We screened six aminoglycosides, namely, gentamicin sulfate (Sigma, Cat# G1914), neomycin sulfate (Sigma, Cat# PHR1491), streptomycin sulfate (Sigma, Cat# S6501), tobramycin sulfate (Sigma, Cat# T1783), kanamycin sulfate (Sigma, Cat# 60615), and kanamycin disulfate (Sigma, Cat# K1876). 100 μl of 10 mg/ml aqueous solutions of antibiotics, 100 μl of deionized (DI) water, and either 100 μl of algae extract or 100 μl of 10 mg/ml sodium alginate (Sigma, Cat# W201502) were mixed together to obtain a total reaction volume of 300 μl in each well of 96-well microtiter plates. To vary the concentration of antibiotics, the subsequent wells were filled with 80 μl, 60 μl, 40 μl, 20 μl, and 10 μl of 10 mg/ml antibiotic stock solution with the remaining volume out of 300 μl reactions compensated by DI water. Optical density at 600 nm (OD600) at room temperature was measured using a plate reader (BioTek, Model# Synergy2-Cam4, Software-Gen5-1.08) (Fig 1a). A Nikon Coolpix camera was used to image wells in microtiter plates (Fig 1b).

### Measurement of melting temperature of alginate polymer

Sodium alginate (20 mg/ml) was mixed with kanamycin disulfate (50 mg/ml) in a volume ratio of 1:1 ratio (10 ml each). Resultant solution with alginate polymer was centrifuged at 10,000 rpm for 10 min. The supernatant was discarded and the pellet was washed three times with 5 ml of sterile 0.1 M (pH 7.4) phosphate-buffered saline (PBS) buffer. Six pellets were made and incubated with 2 ml of 1 M NaOH at 10°, 20°, 40°, 60°, 80° and 100°C for 30 min. After incubation, tubes were centrifuged at 10000 rpm for 10 min and absorption spectra of supernatants (Fig 2) were measured using a spectrophotometer (Thermo Scientific Evolution 260 Bio Spectrophotometer).

### Imaging and analysis of alginate polymer texture using light microscope

100 μl of 10 mg/ml aqueous solutions of antibiotics were mixed drop-wise with either 100 μl of algae extract or 100 μl of 10 mg/ml sodium alginate on a clean glass slide. The immediate polymerization reaction was observed and imaged using a light microscope at 10X magnification (Fig 3). To describe the polymer texture quantitatively, we converted the images into 8-bit gray scale images with 256 gray levels. Using the gray scale values, *i*, we determined four quantitative parameters of the Gray Level Co-Occurrence Matrix (GLCM) using the ImageJ Texture Analyzer plugin[61]: the energy, *E* = ∑*p*(*i*, *j*)^2^; the contrast, *C* = ∑|*i* − *j*|^2^ *p*(*i*, *j*); the homogeneity, *H* = ∑*p*(*i*, *j*) / (1 + |*i* − *j*|); the entropy, *S* = −∑*p*(*i*, *j*)log[*p*(*i*, *j*)]; where the sums are for all distinct gray scale values and *p*(*i*, *j*) is the (*i*, *j*)*th* element of the normalized gray scale spatial dependence matrix. Three images were chosen for each biopolymer, converted to 8-bit images, and analyzed with step size of 1 pixel at 0° angle. From the same 8-bit images, the fractal dimension was measured using the FracLac plugin for ImageJ by using the default settings of the FracLac program [62]. All five parameters with standard deviations are given in Table 1.

### Scanning Electron Microscopy (SEM) of alginate polymer

Biopolymer was made by mixing sodium alginate and kanamycin disulfate in a volume ratio of 1:1. The polymer suspension was spread on a clean glass slide and imaged using Phenom Pro-Scanning Electron Microscope (Fig 4). It should be noted that drying can lead to a loss of polymer flexibility and therefore, should be imaged quickly.

### Quantitative weight-based assay of polymer formation

To quantify polymer formation, 500 μl of either 10 mg/ml aqueous sodium alginate solution or aqueous algae extract was added to a microcentrifuge tube and antibiotics were added at varying final concentrations. The reactions were vortexed and incubated for 10 min at room temperature. After incubation, tubes were centrifuged at 10000 rpm for 15 min and polymer pellets were weighed using an analytical balance. Weight of polymer pellets in mg as a function of aminoglycoside concentrations in mg/ml were plotted (Fig 5), which were fitted to a model that also described the growth of pathogen prion protein growth [45]. Fit parameters are given in Table 2.

### Antimicrobial activity of alginate polymer

Antimicrobial activity of polymers was checked against *E*. *coli* DH5α using agar diffusion assay [63]. Single colony of *E*. *coli* was inoculated in 10 ml of Luria Broth (LB) and incubated till OD600 reached 0.5 (log-phase culture). 100 μl of bacterial culture was spread over LB agar plates. To check the antimicrobial activity of the antibiotics, wells were made by punching a hole in LB agar plates. For reference plates, 50 μl of 10 mg/ml aqueous stock solution of each antibiotic was added in the wells (Fig 6, top row). For experiments with alginate polymers, polymer pellets were washed 3 times with sterile DI water to remove any unbound free antibiotics. Each washing step included the addition of 500 μl of sterile DI water and centrifugation at 10000 rpm for 15 min. After washing, polymer pellets were placed at the center of the LB plates with *E*. *coli* DH5α. Plates were incubated at 37°C for 18 hr. Zone of inhibition, i.e., the area with no growth of bacteria around well or polymer pellet was measured using a ruler (Fig 6, middle and bottom rows; Table 3).

### Biocompatibility assay

We used COS-1 cell lines to test biocompatibility of antimicrobial alginate polymer. Sterile stock solutions of sodium alginate (10 mg/ml) and kanamycin disulfate (100 mg/ml) were mixed in a volume ratio of 1:1 in microtiter plate wells under sterile conditions. After 30 min incubation at 20°C, thin biopolymer layers were visible at the bottom of microtiter wells. We washed wells three times with 2 ml of sterile 0.1 M (pH 7.4) PBS buffer to remove unbound polymer. 1.5 ml of culture stock of COS-1 cells were added to the wells under sterile condition and incubated for 3 days at 37°C at 5% CO_2_ environment. After incubation, plates were imaged before and after staining with methylene blue using a light microscope and analyzed using ImageJ (Fig 7).
